# The Photosynthesis, Na^+^/K^+^ Homeostasis and Osmotic Adjustment of *Atriplex canescens* in Response to Salinity

**DOI:** 10.3389/fpls.2016.00848

**Published:** 2016-06-17

**Authors:** Ya-Qing Pan, Huan Guo, Suo-Min Wang, Bingyu Zhao, Jin-Lin Zhang, Qing Ma, Hong-Ju Yin, Ai-Ke Bao

**Affiliations:** ^1^State Key Laboratory of Grassland Agro-ecosystems, College of Pastoral Agriculture Science and Technology, Lanzhou UniversityLanzhou, China; ^2^Department of Horticulture, Virginia Polytechnic Institute and State UniversityBlacksburg, VA, USA

**Keywords:** *Atriplex canescens*, salt tolerance, photosynthesis, osmotic adjustment, salt bladder

## Abstract

*Atriplex canescens* (fourwing saltbush) is a C_4_ perennial fodder shrub with excellent resistance to salinity. However, the mechanisms underlying the salt tolerance in *A. canescens* are poorly understood. In this study, 5-weeks-old *A. canescens* seedlings were treated with various concentrations of external NaCl (0–400 mM). The results showed that the growth of *A. canescens* seedlings was significantly stimulated by moderate salinity (100 mM NaCl) and unaffected by high salinity (200 or 400 mM NaCl). Furthermore, *A. canescens* seedlings showed higher photosynthetic capacity under NaCl treatments (except for 100 mM NaCl treatment) with significant increases in net photosynthetic rate and water use efficiency. Under saline conditions, the *A. canescens* seedlings accumulated more Na^+^ in either plant tissues or salt bladders, and also retained relatively constant K^+^ in leaf tissues and bladders by enhancing the selective transport capacity for K^+^ over Na^+^ (ST value) from stem to leaf and from leaf to bladder. External NaCl treatments on *A. canescens* seedlings had no adverse impact on leaf relative water content, and this resulted from lower leaf osmotic potential under the salinity conditions. The contribution of Na^+^ to the leaf osmotic potential (Ψ*s*) was sharply enhanced from 2% in control plants to 49% in plants subjected to 400 mM NaCl. However, the contribution of K^+^ to Ψ*s* showed a significant decrease from 34% (control) to 9% under 400 mM NaCl. Interestingly, concentrations of betaine and free proline showed significant increase in the leaves of *A. canescens* seedlings, these compatible solutes presented up to 12% of contribution to Ψ*s* under high salinity. These findings suggest that, under saline environments, *A. canescens* is able to enhance photosynthetic capacity, increase Na^+^ accumulation in tissues and salt bladders, maintain relative K^+^ homeostasis in leaves, and use inorganic ions and compatible solutes for osmotic adjustment which may contribute to the improvement of water status in plant.

## Introduction

Salinity is one of the major environmental factors reducing the growth, development, and productivity of plants (Zhu, 2001; Flowers, 2004; Zhang et al., 2010; Shabala, 2013; Yan et al., 2013; Tang et al., 2015; Kalaji et al., 2016). It is estimated that about 10% of land area and half of irrigated land in the world are affected by salinity (Ruan et al., 2010; Shabala, 2013). Salt stress adversely reduces plant growth through ionic toxicity and osmotic stress (Hasegawa et al., 2000; Tester and Davenport, 2003; Flowers and Colmer, 2008), which influence a series of physiological processes, and finally suppress the photosynthesis (Zhu, 2001; Flowers and Colmer, 2008; Zhang and Shi, 2013; Flowers et al., 2015; Zhang et al., 2016). Some plants, such as halophytes, however, have evolved multiple adaptive strategies that ensure their survival and growth in a harsh environment (Flowers, 2004; Shabala, 2013). Therefore, to cope with the challenge of salinity for agriculture, there are increasing interests of studying the physiological responses underlying the salt resistance of halophytes, especially those species with high economic value and salt tolerance (Yamaguchi and Blumwald, 2005; Yan et al., 2013).

Salt resistance is a complex trait involving multiple mechanisms. One of the effective adaptations is reduction of Na^+^ concentration in cytosol to alleviate Na^+^ toxicity and maintain the intracellular ion homeostasis in a saline environment. Halophytes do achieve this goal by controlling net Na^+^ uptake in the root, excreting Na^+^ from the surface of stem or leaf, and sequestering Na^+^ into the vacuole (Zhu, 2003; Flowers and Colmer, 2008; Hasegawa, 2013). Some species, such as *Thellungiella halophila* (Volkov and Amtmann, 2006; Wang et al., 2006) and *Puccinellia tenuiflora* (Wang et al., 2009, 2015; Guo et al., 2012; Niu et al., 2016) maintain a high K^+^/Na^+^ ratio in shoots by limiting net Na^+^ influx into roots. Other species take up Na^+^ from soil and then excrete large quantities of Na^+^ via salt glands or bladders (Flowers and Colmer, 2008; Ding et al., 2010; Shabala, 2013; Shabala et al., 2014). Some succulent halophytes can sequester Na^+^ into the vacuole to reduce the toxicity of excessive Na^+^ in cytosol as well as regulate cellular osmotic potential by using Na^+^ as an osmoregulation substance, and thus maintain cellular ion homeostasis and turgor (Zhu, 2001; Flowers, 2004; Wang et al., 2004, 2007; Zörb et al., 2005; Yamaguchi et al., 2013; Flowers et al., 2015). The osmotic adjustment (OA) is another important physiological mechanism for plant adaptation to salinity, which involves the fall of osmotic potential (Ψ*s*) in plant tissue resulting from the net accumulation of cellular solutes. It is essential for plants to maintain water uptake from a low water potential environment (Zhang et al., 1999; Munns and Tester, 2008; Ma et al., 2012). Inorganic ions, such as K^+^ and vacuolar Na^+^, can directly be engaged in decreasing the osmotic potential of cells (Flowers and Colmer, 2008). Moreover, higher plants also accumulate compatible solutes, such as betaine, free proline and soluble sugars, for OA under abiotic stress (Munns and Tester, 2008; Flowers et al., 2010).

*Atriplex canescens* (Pursh) Nutt. (fourwing saltbush), a C_4_ perennial shrub native to saline and xeric deserts in North America, belongs to Chenopodiaceae with prominent resistance to salinity, drought, and cold (Glenn and Brown, 1998; Hao et al., 2013). This species is also an attractive fodder shrub for most livestock and large animals due to its high palatability as well as rich nutrition (Peterson et al., 1987; Kong, 2013). Moreover, it is especially useful for erosion control and reclamation of marginal lands due to its extensive root system and excellent adaptability (Benzarti et al., 2013; Kong, 2013). In 1989, *A. canescens* was introduced from USA to China and was widely used for soil and water conservation, sand-fixing and saline land restoration in north China (Kong, 2013). Previous studies showed that *A. canescens* accumulated more Na^+^ under salinity conditions (Glenn et al., 1994, 1996; Glenn and Brown, 1998). On the other hand, recent studies suggested that the salt excretion via salt bladders (Ben Hassine et al., 2009; Belkheiri and Mulas, 2011; Shabala et al., 2014; Tsutsumi et al., 2015), the uptake and accumulation of K^+^ (Bazihizina et al., 2009; Bose et al., 2015), photosynthetic responses (Redondo-Gómez et al., 2007; Bazihizina et al., 2009; Nemat-Alla et al., 2011), and OA by compatible solutes (Martínez et al., 2004, 2005; Ben Hassine et al., 2008; Bouchenak et al., 2012), may also contribute to the salt tolerance in some species of *Atriplex*. However, it is not clear if similar or different physiological mechanisms also contribute to the responses of *A. canescens* to saline environment.

Therefore, the aim of this study was to characterize the physiological responses of *A. canescens* to salinity by measuring various parameters related to photosynthesis, Na^+^/K^+^ homeostasis and OA under treatments with different concentrations of NaCl.

## Materials and Methods

### Plant Growth Conditions and NaCl Treatments

Seeds of *A. canescens* were collected in Lingwu County (37.78° N, 106.25° E; elevation 1250 m) of Ningxia Autonomous Region, China. After corrosion of the hard coat with 75% H_2_SO_4_ (v/v) for 15 h, seeds were rinsed six times with distilled water and, germinated in vermiculite (moistened with distilled water) at 28°C in the dark for 6 days. Uniform seedlings were transplanted to plastic culture pots (5 cm × 5 cm × 5 cm; two plants/pot) containing vermiculite (with trace amounts of Na^+^ and K^+^, Ma et al., 2012) and watered with 1/2 strength Hoagland nutrient solution (Ma et al., 2012) at 2-days interval. The growth conditions in greenhouse were controlled to maintain a temperature of 28°C/25°C (day/night), a photoperiod of 16/8 h (light/dark; the flux density was about 800 μmol m^-2^ s^-1^) and an approximate relative humidity of 65%.

After washing the leaves thoroughly with distilled water (to remove the salt from the surface of the leaves), 5-weeks-old seedlings were treated with 1/2 strength Hoagland nutrient solution containing additional 0, 100, 200, or 400 mM NaCl for 10 days, and the treatment solutions were renewed every 2 days to keep constant NaCl concentration. The treated and control plants were harvested for biomass measurement and physiological analysis.

### Measurement of Parameters for Photosynthesis and Water Relations

Net photosynthesis rate (Pn), stomatal conductance (Gs), and transpiration rate (Tr) were measured by an automatic photosynthetic measuring apparatus (GFS-3000; Walz, Effeltrich, Germany) in the greenhouse under a light intensity of 900 μmol m^-2^ s^-1^. The water use efficiency (WUE) was calculated by the following formula: WUE = Pn/Tr (Ma et al., 2012). Leaf areas were estimated using a photo scanner (Epson Perfection 4870; Epson America, Inc., Long Beach, CA, USA).

### Measurement of Na^+^ and K^+^ Concentrations

Salt bladders were brushed from both adaxial and abaxial surfaces of leaves (approximately 1 g of fresh weight of leaves was used) into 20 mL deionized water using a hard nylon brush as described by Tsutsumi et al. (2015). Collected leaves were dried in an oven at 80°C for 72 h, and the dry weight (DW) was determined. Roots were rinsed with deionized water for 10 s, washed twice for 8 min in cold 20 mM LiNO_3_ solution to exchange the cations in the apoplast. The DWs of stems and roots were determined after drying at 80°C for 72 h.

For determining the cation exclusion in bladders, the Na^+^ and K^+^ were extracted from brushed bladders under 90°C water bath for 1 h. The Na^+^ and K^+^ were determined using a flame photometer (Model 410 Flame; Sherwood Scientific, Ltd., Cambridge, UK), and cation concentration in salt bladders was calculated by the following formula: cation (Na^+^ or K^+^) concentration in salt bladders (mmol/g DW) = cation content in salt bladders (mmol)/ DW (g) of leaves. For measuring the cation accumulation in tissues, the Na^+^ and K^+^ were extracted from dried roots, stems and leaves with 100 mM acetic acid at 90°C for at least 2 h, the cation concentrations were then determined using flame spectrophotometer (Bao et al., 2016).

Selective transport (ST) capacity for K^+^ over Na^+^ between different parts (root, stem, leaf, and bladder) was calculated according to the following equation (Wang et al., 2002, 2009): ST_(A/B)_ = (Na^+^/K^+^ in part A)/(Na^+^/K^+^ in part B). The higher ST value indicates the stronger net capacity of selection for transport of K^+^ over Na^+^ from part A to part B (Wang et al., 2002; Ma et al., 2014).

### Scanning Electron Microscopic Observation of Leaf Surface

Segments of leaves before and after brushing the salt bladders were fixed on a stainless steel bracket and frozen with liquid nitrogen, then the samples were taken out the liquid nitrogen and the abaxial surfaces of leaves were observed quickly using scanning electron microscope (S-3400N; Hitachi, Tokyo, Japan). Meanwhile, the images were taken. The accelerating voltage was 15 kV.

### Measurement of Betaine and Free Proline Concentration

For betaine determination, mature leaves from plants with different treatments were dried at 80°C for 1 day and ground to pass a 40-mesh sieve. The dried, finely ground sample (0.2 g) was shaken with 1 mL of 80% methanol (v/v) at 60°C for 30 min. The extracted solution was harvested after centrifugation at 11,000 × *g* under 25°C for 15 min. Then the betaine concentration was measured with a Reinecke salt Kit (Comin Biotechnology, Co. Ltd., Suzhou, China) following the manufacturer’s instructions. Briefly, 0.25 mL of the extracted solution was mixed with 0.35 mL of Reinecke salt saturated solution (30 mg/L, pH = 1.0) and the reaction proceeded at 4°C for 2 h. The supernatant was discarded after centrifugation at 10,000 × *g* under 25°C for 15 min. The precipitate was washed with 0.3 mL of 99% ether (v/v) and then dissolved in 1 mL of 70% acetone (v/v). Finally, the absorbance was measured at 525 nm using a spectrophotometer (UV-6100PCS; Mapada Instruments, Co. Ltd., Shanghai, China). The betaine concentration was calculated in comparison with a standard sample in Kit.

For free proline measurement, 0.1 g of fresh leaf was homogenized with 1 mL of 5% salicylic acid (v/v) on the ice, then was extracted with shaking in boiling water for 10 min. The supernatant was collected after centrifugation at 10,000 × *g* under 25°C for 10 min. Finally, free proline concentration was determined according to the method described by Wang et al. (2004) using a spectrophotometer.

### Measurement of Leaf Relative Water Content

The leaf relative water content (RWC) was calculated according to the following formula: RWC (%) = 100 × (FW - DW)/(TW - DW) (Bao et al., 2016). The leaves were excised from seedlings and the fresh weight (FW) was weighed immediately, then the turgid weight (TW) was measured after soaking the leaves in deionized water at 4°C overnight in the dark. Finally, leaves were dried in an oven at 80°C for 48 h and the DW were determined.

### Measurement of Leaf Osmotic Potential (Ψ*s*) and Evaluation of the Contributions of Solutes to Leaf Ψ*s*

Leaves from each treatment were rinsed with deionized water and blotted on filter paper immediately, then were frozen in liquid nitrogen and thawed to extrude sap by a syringe, respectively. The resulting sap was used to determine the leaf osmotic potential (Ψ*s*) according to the method described by Bao et al. (2014) using a cryoscopic osmometer (Osmomat-030, Gonotec GmbH, Berlin, Germany) at 25°C. To evaluate the contributions of solutes to leaf Ψ*s*, the calculated osmotic potential (COP) values of Na^+^, K^+^, betaine, and free proline were calculated respectively by the Van’t Hoff equation (Guerrier, 1996): COP = moles of solute × RK, where *R* = 0.008314 and *K* = 298°C. Then the contribution of each solute to leaf osmotic potential (C) was calculated by the following formula: C = COP/Ψs × 100% (Guerrier, 1996).

### Statistical Analysis

Data were analyzed according to one-way analysis of variance (ANOVA) by SPSS statistical software (Ver. 19.0, SPSS, Inc., Chicago, IL, USA) and the significant differences among means were identified by Duncan’s multiple range tests at a significance level of *P* < 0.05. All data were presented as mean ± SE (*n* ≥ 8).

## Results

### *Atriplex canescens* Seedlings Exhibited Strong Resistance to Salinity

**FIGURE 1:**
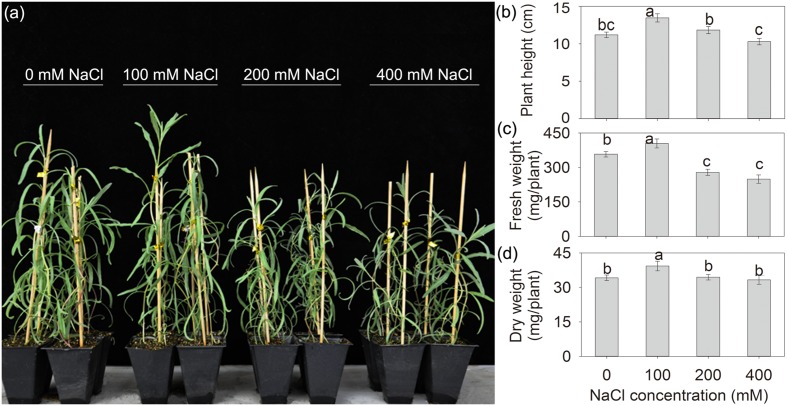
**The growth status (a), plant height (b), fresh weight (c), and dry weight (d) of *Atriplex canescens* seedlings under different NaCl treatments for 10 days.** Values in **(b–d)** are mean ± SE (*n* = 17–23). Columns with different letters indicate significant differences at *P <* 0.05 (Duncan test).

After treatment with various concentrations of external NaCl for 10 days, all of the seedlings grew vigorously, especially, the seedlings under 100 mM NaCl exhibited larger and sturdier phenotypes than those under control (0 mM NaCl) and other NaCl treatments (**Figure 1a**), indicating that the addition of 100 mM NaCl might promote the growth of *A. canescens*. To further confirm above observations, plant height and biomass were measured. The data showed that the addition of 100 mM NaCl significantly increased plant height, FW and DW of *A. canescens* seedlings by 20, 13, and 15%, respectively, compared to control plants (**Figures 1b–d**). Furthermore, compared with the control, the addition of either 200 or 400 mM NaCl had no significant negative effects on plant height (**Figure 1b**) and DW (**Figure 1d**), although significantly reduced FW of plants (**Figure 1c**).

### Effects of External NaCl on Photosynthesis of *A. canescens* Seedlings

**FIGURE 2:**
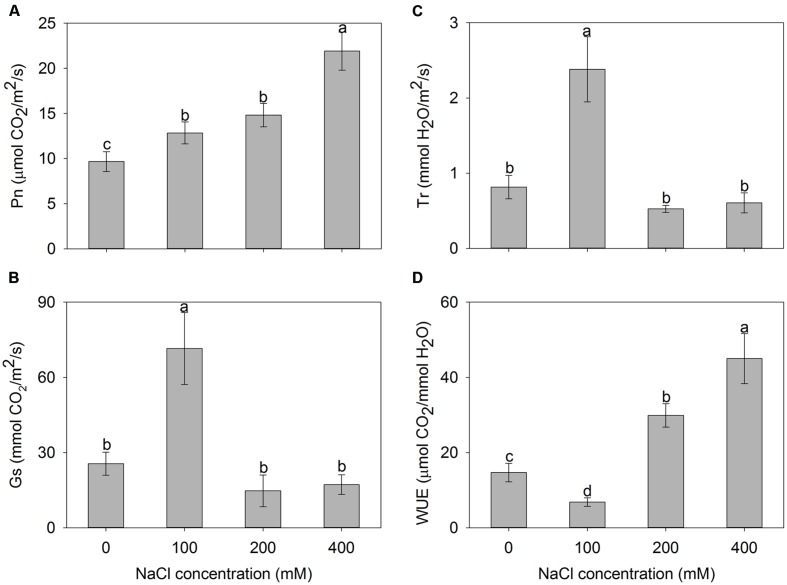
**Net photosynthesis rate (Pn) (A), stomatal conductance (Gs) (B), transpiration rate (Tr) (C), and water use efficiency (WUE) (D) of *A. canescens* seedlings under different NaCl treatments for 10 days.** Values are mean ± SE (*n* = 10). Columns with different letters indicate significant differences at *P <* 0.05 (Duncan test).

To investigate the photosynthetic capacity of *A. canescens* seedlings under saline conditions, the net photosynthetic rate (Pn), stomatal conductance (Gs), transpiration rate (Tr), and WUE were measured. The results showed that the Pn of plants under NaCl treatments were significantly higher than that of control plants, and it actually increased within the measured range of NaCl concentrations. After 10 days of treatment, Pn of plants exposed to 100, 200, and 400 mM NaCl were 1.3, 1.5, and 2.3 fold higher than of control plants, respectively (**Figure 2A**). Compared to control, interestingly, Gs and Tr of *A. canescens* seedlings in the presence of additional 100 mM NaCl showed a sharp increase by 180 and 190%, respectively. However, both Gs and Tr were unaffected by 200 or 400 mM NaCl (**Figures 2B,C**). Correspondingly, the plant WUE was significantly reduced under 100 mM NaCl, but increased in the presence of 200 and 400 mM NaCl, which are 2.1 and 3.1 fold higher than for control plants, respectively (**Figure 2D**).

### The Na^+^/K^+^ Homeostasis in *A. canescens* Seedlings Exposed to Salinity

To investigate the mechanism underlying salt resistance of *A. canescens* seedlings, we measured the amounts of Na^+^ and K^+^ accumulated in tissues and sequestered in salt bladders respectively, and also estimated the ST capacity for K^+^ over Na^+^ between different parts of *A. canescens* seedling.

**FIGURE 3:**
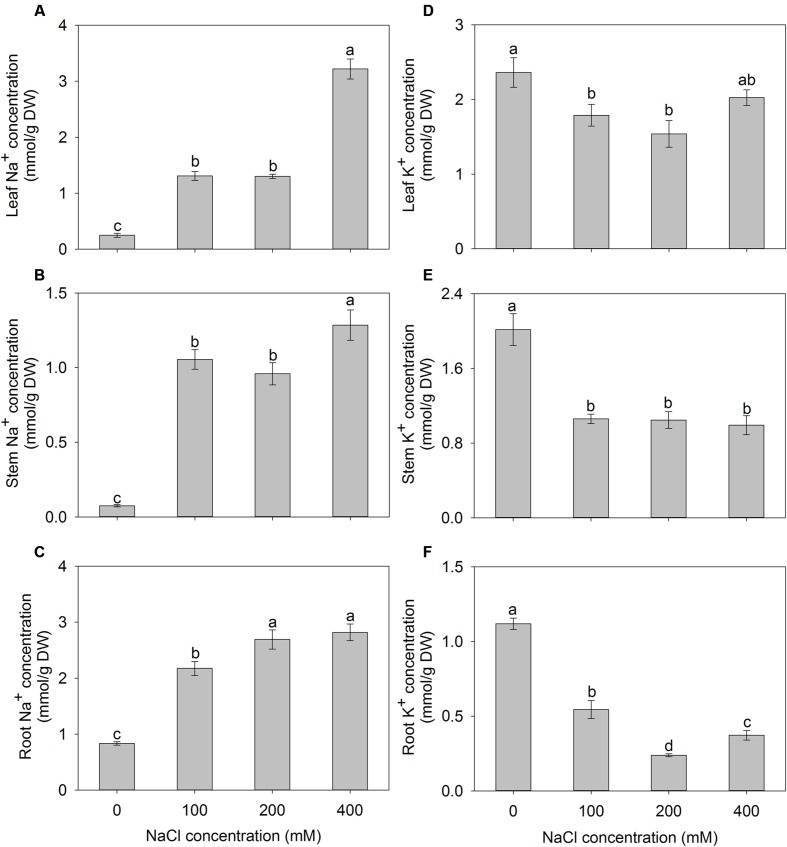
**The Na^+^ (A–C) and K^+^ (D–F) concentrations in the leaves, stems and roots of *A. canescens* seedlings under different NaCl treatments for 10 days.** Values are mean ± SE (*n* = 10). Columns with different letters indicate significant differences at *P <* 0.05 (Duncan test).

With the increase of the external NaCl concentration, Na^+^ accumulation exhibited a significant increase in different tissues of *A. canescens* seedlings. When treated with 400 mM NaCl for 10 days, the Na^+^ concentrations in leaves, stems, and roots were 13.4, 17.2, and 3.4 fold higher than those in control plants, respectively (**Figures 3A–C**). Although, K^+^ accumulations in all tissues of *A. canescens* seedlings were reduced by external NaCl (**Figures 3D–F**), the K^+^ concentration in stems maintained a relative stability among all external NaCl treatments (**Figure 3E**), and especially in leaves, it showed lesser decrease by 24 and 35% under 100 and 200 mM NaCl compared to control plants, respectively, even rebounded to control level under 400 mM NaCl (**Figure 3D**).

**FIGURE 4:**
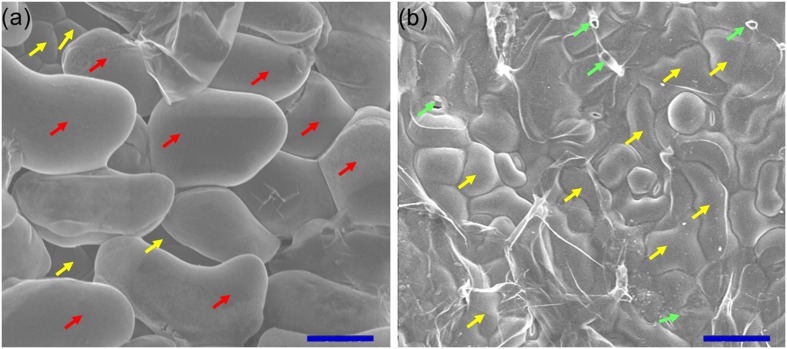
**Scanning electron microscopic observation of leaf surface.** Before **(a)** and after **(b)** brushing the salt bladders, the abaxial surfaces of leaves were observed and photographed using scanning electron microscope and the images were taken subsequently. The accelerating voltage was 15 kV. Red arrows, salt bladders; yellow arrows, epidermal cells; green arrows, stalk cell. Bar = 0.1 mm.

**FIGURE 5:**
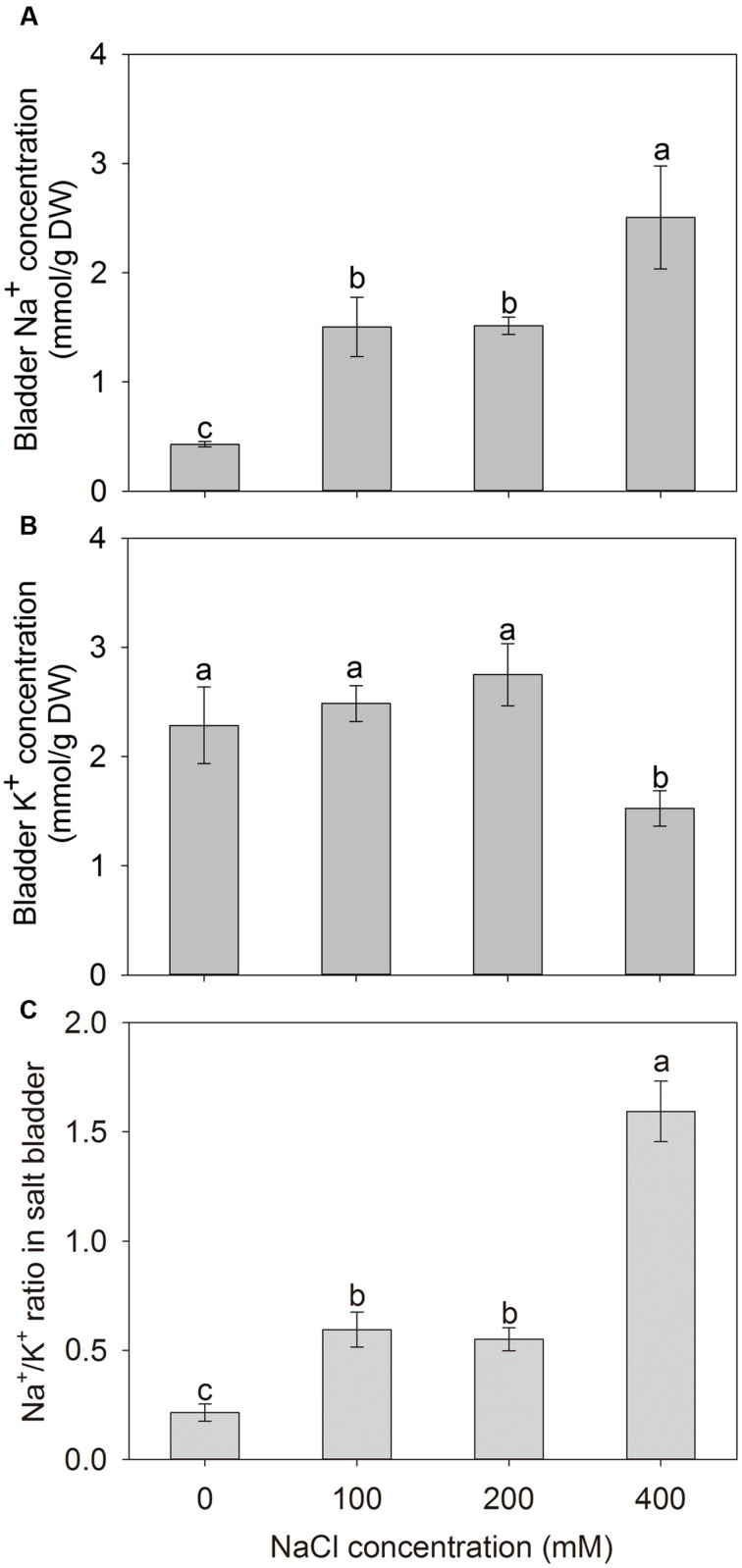
**The Na^+^ (A) and K^+^ (B) concentrations, and Na^+^/K^+^ ratio (C) in salt bladders of *A. canescens* seedlings under different NaCl treatments for 10 days.** Values are mean ± SE (*n* = 10). Columns with different letters indicate significant differences at *P <* 0.05 (Duncan test).

To investigate the Na^+^ sequestration in salt bladders of *A. canescens* seedlings, we brushed the salt bladders from the surface of leaves (**Figure 4**). When *A. canescens* seedlings were grown in normal conditions (without NaCl supplement), only a small amount of Na^+^ was measured in salt bladders. However, the bladder Na^+^ concentration significantly raised with the increasing of external NaCl. Under 100, 200, and 400 mM NaCl for 10 days, the bladder Na^+^ concentrations were 3.5, 3.6, and 5.9 fold higher than that of control, respectively (**Figure 5A**). On the other hand, compared to control, the bladder K^+^ concentration was unaffected by 100 and 200 mM NaCl, and was significantly reduced by 33% under 400 mM NaCl (**Figure 5B**). Correspondingly, more Na^+^ accumulation resulted in a significant increase of Na^+^/K^+^ ratio in salt bladders under various NaCl treatments. For example, the bladder Na^+^/K^+^ ratio of *A. canescens* seedlings under 400 mM NaCl (the value is 1.6) was 7.4 fold higher than that of the control plants (the value is 0.2; **Figure 5C**). These results indicate that sequestering more Na^+^ into bladder may be one of important strategies for *A. canescens* seedlings to alleviate the toxicity of excessive Na^+^ under saline conditions.

**FIGURE 6:**
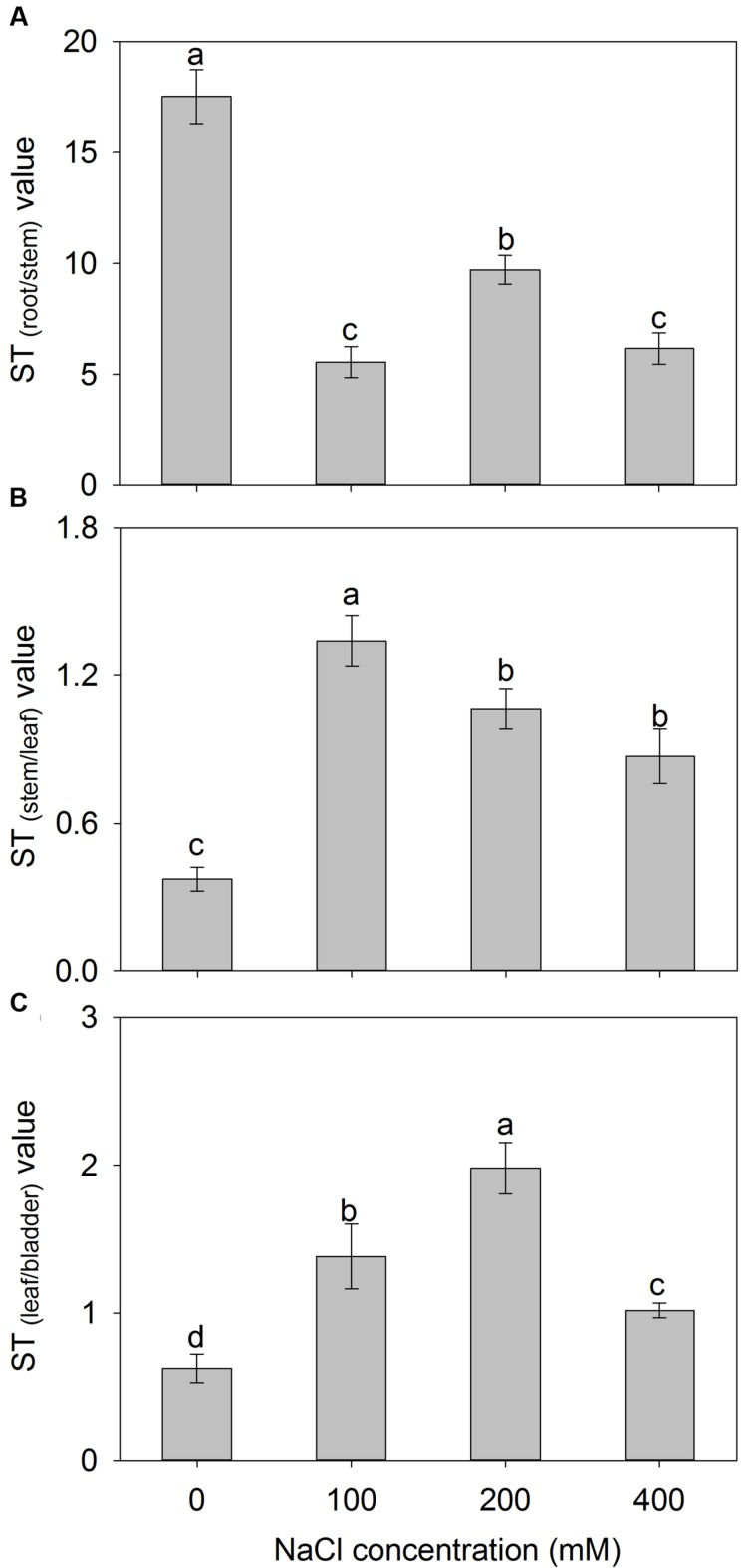
**The selective transport capacity for K^+^ over Na^+^ (ST value) from root to stem (A), from stem to leaf (B) and from leaf to salt bladder (C) in *A. canescens* seedlings under different NaCl treatments for 10 days.** ST_(A/B)_ value = (Na^+^/K^+^ in part A)/(Na^+^/K^+^ in part B). Values are mean ± SE (*n* = 10). Columns with different letters indicate significant differences at *P <* 0.05 (Duncan test).

The addition of external NaCl also influenced the ST capacity for K^+^ over Na^+^ (ST value) in *A. canescens* seedlings, but the change patterns of ST value varied among different parts (**Figure 6**). Compared to control, the ST values from root to stem were significantly decreased by 68, 45, and 65% under 100, 200, and 400 mM NaCl, respectively (**Figure 6A**). However, the ST values from stem to leaf of plants treated with 100–400 mM NaCl were significantly higher by 2.6, 1.8, and 1.3 fold than that in control plants, respectively (**Figure 6B**). Interestingly, the ST value from leaf to bladder showed significant increase by 1.2, 3.2, and 0.6 fold under 100, 200, 400 mM NaCl, respectively (**Figure 6C**). Most importantly, *A. canescens* seedlings showed highest ST value from root to stem under either control or saline conditions, suggesting that relatively more K^+^ may be selectively loaded to xylem (**Figure 6**).

### *A. canescens* Seedlings Accumulated More Betaine and Free Proline during NaCl Treatment

**FIGURE 7:**
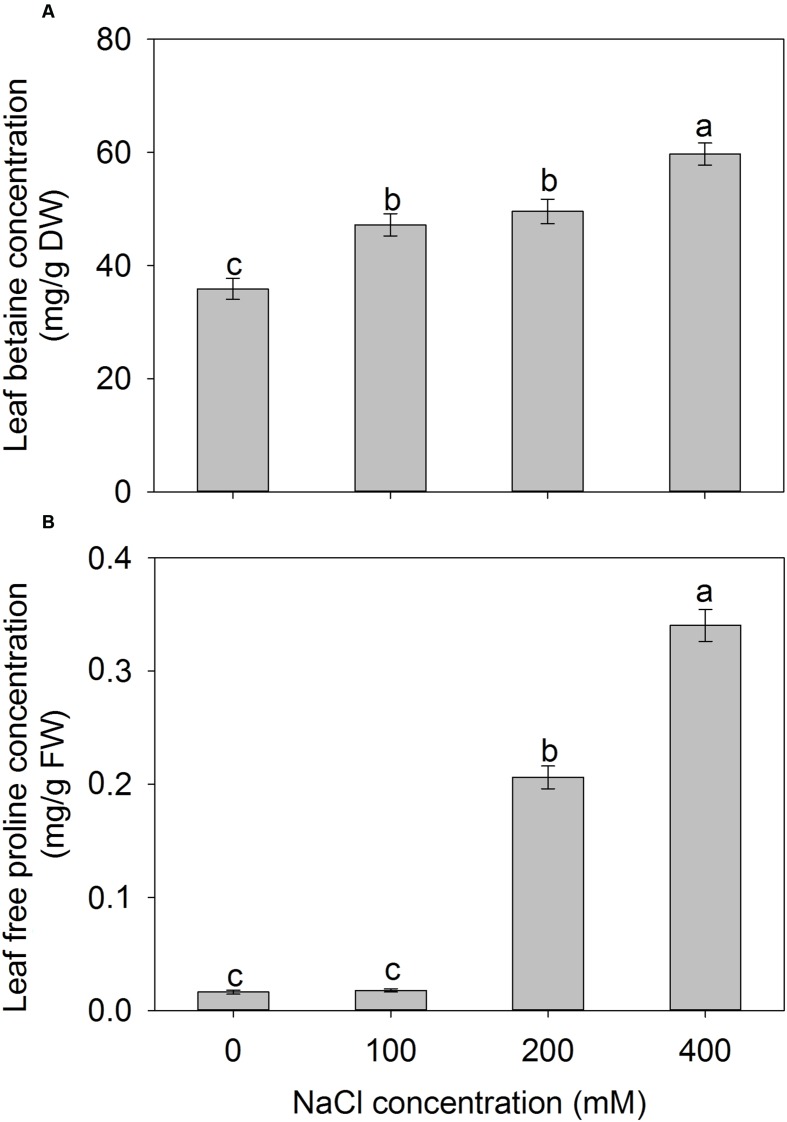
**The betaine (A) and free proline (B) concentrations in leaves of *A. canescens* seedlings under different NaCl treatments for 10 days.** Values are mean ± SE (*n* = 8). Columns with different letters indicate significant differences at *P <* 0.05 (Duncan test).

To investigate the effect of NaCl on compatible solute in *A. canescens* seedlings. We measure the concentrations of betaine and free proline in leaves. The leaf betaine concentration gradually increased with the increase of external NaCl concentration, and the highest value was detected under 400 mM NaCl, which was 66% higher than that of control plants (**Figure 7A**). Moreover, the leaf free proline concentrations of *A. canescens* seedlings were very low under either control or 100 mM NaCl conditions, but were sharply enhanced by 12 and 20 fold under 200 and 400 mM NaCl than in control, respectively (**Figure 7B**). These results indicate that salinity (especially at a high concentration) can induce more accumulation of compatible solutes in *A. canescens* seedlings.

### *A. canescens* Seedlings Maintain Higher Leaf Relative Water Content by Effective Osmotic Adjustment Under Salinity Conditions

**FIGURE 8:**
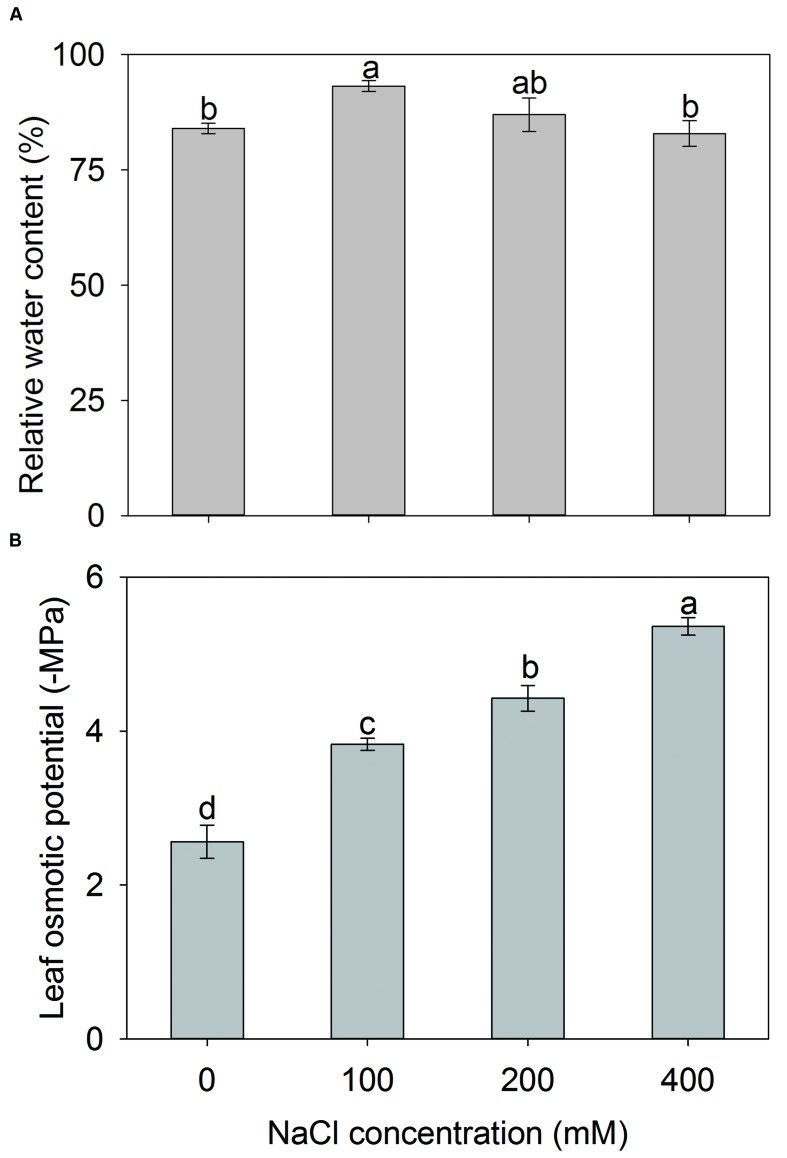
**Relative water content (A) and osmotic potential (B) in leaves of *A. canescens* seedlings under different NaCl treatments for 10 days.** Values are mean ± SE (*n* = 8). Columns with different letters indicate significant differences at *P <* 0.05 (Duncan test).

Maintaining water balance in plants is essential for their survival under saline conditions. Therefore, the leaf RWCs were determined for *A. canescens* seedlings after NaCl treatment. Compared with control, leaf RWC of *A. canescens* seedlings was not reduced by additional NaCl regardless of the concentrations, even showed a significant increase of 11% under 100 mM NaCl treatment (**Figure 8A**). These results are consistent with the growth data (**Figure 1**) and it implies that water status in plant may be one of key factors contributing to the survival and development of *A. canescens* seedlings under saline conditions.

To investigate the mechanism underlying high water retention capacity in *A. canescens* seedlings, the leaf osmotic potential (Ψ*s*) was measured. As shown in **Figure 8B**, the addition of NaCl significantly decreased the leaf Ψ*s* of *A. canescens* seedlings. The leaf Ψs continuously decreased in response to the increase of NaCl concentrations, which suggests that *A. canescens* seedlings could maintain a higher OA capacity in response to salinity. Finally, the contributions of different solutes in leaves to Ψ*s* were further evaluated. With the increase of external NaCl concentrations, the contribution of Na^+^ to Ψ*s* significantly increased from 2% in control plants to 32, 35, and 49% in plants treated with 100, 200, and 400 mM NaCl, respectively. However, the contribution of K^+^ significantly dropped from 34% in control plants to 9% in plants under 400 mM NaCl (**Table 1**). The contributions of both betaine and free proline showed significant increases under high concentration of NaCl treatments (200 and 400 mM) and accounted for 8 and 4% of contributions to Ψ*s* under 400 mM NaCl, respectively (**Table 1**).

**Table 1:** The contributions of Na^+^, K^+^, betaine, and free proline to leaf osmotic potential (Ψ*s*) of *Atriplex canescens* seedlings under different NaCl treatments for 10 days.

NaCl treatments (mM)	Contribution of Na^+^ to Ψ*s* (%)	Contribution of K^+^ to Ψ*s* (%)	Contribution of betaine to Ψ*s* (%)	Contribution of free proline to Ψ*s* (%)
0	2.0 ± 0.3c	33.8 ± 5.2a	6.0 ± 0.3b	0.3 ± 0.1c
100	32.4 ± 3b	26.0 ± 3.2ab	6.4 ± 0.3b	0.3 ± 0.1c
200	34.7 ± 5.5b	19.9 ± 2.4b	7.3 ± 0.4a	2.8 ± 0.2b
400	48.8 ± 4.2a	9.2 ± 1.1c	8.1 ± 0.3a	3.8 ± 0.2a

*Values are mean ± SE (n = 8). Columns with different letters indicate significant differences at P < 0.05 (Duncan test).*

## Discussion

It was proposed that halophytes such as *Suaeda* spp. grow better at moderate concentrations of NaCl, which is generally harmful to the growth of glycophyte species (Flowers, 2004; Shabala and Mackay, 2011). In the present work, the growth of *A. canescens* seedlings was stimulated by an external 100 mM NaCl. Plant height and biomass were significantly increased under 100 mM NaCl but were unaffected by external 200 or 400 mM NaCl treatments (**Figure 1**). Similar results were reported for other *Atriplex* species, such as *A. halimus* (Bajji et al., 1998; Martínez et al., 2004; Ben Hassine and Lutts, 2010; Nemat-Alla et al., 2011; Bouchenak et al., 2012), *A. gmelini* (Matoh et al., 1987; Tsutsumi et al., 2015), and *A. portulacoides* (Redondo-Gómez et al., 2007). Therefore, *A. canescens* is a typical halophytic species and highly tolerant to salinity.

The growth of higher plants depends directly on the photosynthetic capacity. In the present study, the net photosynthetic rate (Pn) of *A. canescens* was significantly increased by NaCl treatments (**Figure 2A**). However, stomatal conductance (Gs, **Figure 2B**) and transpiration rate (Tr, **Figure 2C**) were unaffected by 200 and 400 mM NaCl, which is different from the findings in a C_3_ xero-halophyte *Zygophyllum xanthoxylum* that showed a positive correlations between Gs and Pn under salinity (Ma et al., 2012). This might be due to the C_4_ properties of *A. canescens*. It was thought that Na^+^ facilitates some biochemical processes in C_4_ pathway photosynthesis such as the conversion of pyruvate into phosphoenolpyruvate (PEP) and the activity of photosystem II (PS II) in mesophyll chloroplasts (Chaves et al., 2011; Kronzucker et al., 2013). Therefore, the Na^+^ might promote the C_4_ photosynthetic process of *A. canescens* seedlings and thus improve the WUE under high salinity (**Figure 2D**).

Maintaining constant intracellular ion homeostasis, especially K^+^ and Na^+^ homeostasis, is essential for a series of physiological processes in living cells, and is more crucial for plant adapting to saline environments (Zhu, 2003; Tang et al., 2015). Glenn et al. (1994, 1996) concluded that the tolerance of *A. canescens* to salinity was due to the accumulation of large amounts Na^+^ in plants. This situation also was found in many species of genus *Atriplex* (Matoh et al., 1987; Bajji et al., 1998; Bose et al., 2015) and other succulent halophytes such as *Suaeda maritima* (Wang et al., 2007; Zhang et al., 2013) and *Z. xanthoxylum* (Wang et al., 2004; Ma et al., 2012, 2016; Yue et al., 2012). However, it has been demonstrated that excessive Na^+^ in the cytosol is deleterious to cell through inhibiting functional enzymes, disrupting acquisition of K^+^, inhibiting K^+^-depending metabolic processes, and causing secondary stresses such as oxidative stress, regardless of species (Maathuis and Amtmann, 1999; Zhu, 2001; Flowers et al., 2015; Volkov, 2015). To reduce cytosolic Na^+^ concentration, some halophytes developed a mechanism of ion compartmentation by sequestering excessive cytosolic Na^+^ into the central vacuole, which alleviates the Na^+^ toxicity, thus maintains ion homeostasis and OA of cell in saline conditions (Zhu, 2003; Yamaguchi et al., 2013; Flowers et al., 2015). In this study, the *A. canescens* seedlings showed less injury (**Figure 1**) although the accumulation of Na^+^ in all tissues of *A. canescens* seedlings showed significant increase under external NaCl treatments (**Figures 3A–C**), suggesting that Na^+^ might be sequestered into the vacuole by the strong capacity of ion compartmentation. This mechanism contributes to maintain a high cytosolic K^+^/Na^+^ ratio, which is one of the most important features that correlated with the salt tolerance of plants, since Na^+^ shares similar physicochemical properties and competes with K^+^ for the binding sites on enzymes in the cytoplasm and other key metabolic processes (Flowers and Colmer, 2008; Shabala and Cuin, 2008). On the other hand, the accumulation of K^+^ was reduced in all tissues of *A. canescens* seedlings by external NaCl (**Figures 3D–F**). Similar results were also observed in many succulent halophytes (Ben Hassine et al., 2009; Nemat-Alla et al., 2011; Shabala and Mackay, 2011; Yue et al., 2012; Bose et al., 2015), and is due to the competition of Na^+^ with K^+^ for uptake into roots (Shabala and Mackay, 2011; Flowers et al., 2015). Interestingly, *A. canescens* seedlings maintained a relatively constant K^+^ concentration in shoots, especially in leaves under saline conditions (**Figures 3D,E**), suggesting that *A. canescens* seedlings might struggle to retain more K^+^ in shoot, especially in leaves, as a result, to maintain a relatively constant cytosolic K^+^/Na^+^ ratio in response to salinity. This conclusion was further supported by the fact that transport capacity for K^+^ over Na^+^ (ST value) from stem to leaf in *A. canescens* seedlings was significantly enhanced by external NaCl treatments (**Figure 5B**), since the higher ST_(A/B)_ value implies the stronger capacity to selectively transport K^+^ over Na^+^ from tissue A to tissue B (Wang et al., 2002; Flowers and Colmer, 2008).

Almost all *Atriplex* species are regarded as salt-excreting plants since these species can sequester large quantities of absorbed Na^+^ into epidermal bladder cells (EBCs) on their leaf surfaces and then release Na^+^ from ruptured EBCs (Flowers and Colmer, 2008; Ding et al., 2010; Shabala, 2013; Shabala et al., 2014). In this study, we found that Na^+^ sequestration in EBCs of *A. canescens* was significantly induced by external NaCl and showed a positive correlationship with the NaCl concentration (**Figures 4** and **5A**). This finding is consistent with the previous studies from other *Atriplex* spp. (Jeschke and Stelter, 1983; Ben Hassine et al., 2009; Tsutsumi et al., 2015), and it was proposed that each EBC could sequester about 1000 fold more Na^+^ compared with leaf cell vacuoles because of its larger volume (Shabala et al., 2014). Similar with the process in ‘traditional’ mesophyll cells, indeed, large quantities of Na^+^ in EBCs are transported into the huge central vacuoles, which will result in cytosolic K^+^ and organic osmolytes accumulating for OA in EBCs (Shabala et al., 2014; Tsutsumi et al., 2015). This viewpoint was supported by our data. Under 100 or 200 mM external NaCl, the K^+^ concentration in EBCs of *A. canescens* seedlings showed no change in comparison with control plants (**Figure 5B**). This may be partly due to the fact that *A. canescens* seedlings maintain high ST capacity for K^+^ over Na^+^ (ST value) from root to stem (**Figure 6A**), as well as increased ST value from leaf to salt bladder under salinity (**Figure 6C**). These results also suggest that there is less selectivity on Na^+^ and K^+^ in salt exclusion via EBC, which is different from the situation in most of other salt-excreting plants with multicellular salt gland such as *Limonium bicolor* (Ding et al., 2010; Feng et al., 2014), *Tamarix ramosissima* (Ma et al., 2011) and *Reaumuria soongarica* (Zhou et al., 2012), which have a high selectivity for the secretion of Na^+^. This implies that there are the different salt-excreting mechanisms between salt bladder and salt gland.

Stable water status is essential for plants to survive from saline conditions. In this study, we found that the RWC in the leaf of *A. canescens* seedlings was increased by 100 mM NaCl, and was unaffected by 200 and 400 mM NaCl (**Figure 8A**), suggesting this species has a high water retention capacity that may result in better growth of plants under salinity. Similar phenotypes were also reported for other *Atriplex* species (Redondo-Gómez et al., 2007; Ben Hassine et al., 2009), and could be explained by decreased osmotic potential of cells (Flowers and Colmer, 2008; Munns and Tester, 2008; Yamaguchi et al., 2013). This viewpoint is supported by measurement of osmotic potential in leaves of *A. canescens* seedlings, which decreased significantly with the increase of external NaCl concentration (**Figure 8B**). Lower osmotic potential results in a higher OA capacity, which facilitates water uptake and thus may maintain the turgor in plants at low water potential conditions.

It is well-known that higher OA in plants subjected to salt stress mainly results from the accumulation of either inorganic ions or compatible solutes (or both; Flowers and Colmer, 2008; Kronzucker et al., 2013). In many cases, however, the importance of each solute to OA is controversial (Glenn et al., 1994, 1996; Bajji et al., 1998; Martínez et al., 2004, 2005; Ben Hassine et al., 2008). Therefore, the contributions of various solutes to OA were investigated under normal (no addition of external NaCl) and saline conditions (100–400 mM NaCl). In control plants, K^+^ accounted for 34% of the leaf osmotic potential that was more than 16 fold higher than Na^+^. The contribution of K^+^ was significantly reduced by NaCl treatments while the contribution of Na^+^ to leaf osmotic potential increased sharply to 49% under 400 mM NaCl (**Table 1**). These results suggest that Na^+^ in mesophyll cell and EBCs of *A. canescens* seedlings can be used as an osmolyte contributing to OA in order to cope with osmotic stress under high salinity. In saline soil, *A. canescens* was able to absorb large quantities of Na^+^ from soil and to accumulate in aboveground tissues (Glenn et al., 1994). Ma et al. (2012) found that *Z. xanthoxylum* can use Na^+^ as an osmoregulatory substance by sequestering Na^+^ in vacuoles of large cells mediated by the tonoplast Na^+^/H^+^ antiporter. In addition to inorganic ions, it was proposed that some compatible solutes, including betaine and free proline, may act as cytoplasmic osmoprotectant involved in OA and/or protection of cellular structures in plants under various abiotic stress conditions (Munns and Tester, 2008; Shabala and Mackay, 2011; Tsutsumi et al., 2015). In the present study, we found that the leaf betaine accumulation of *A. canescens* seedlings positively correlated with the concentration of external NaCl (**Figure 7A**) and its contribution to the leaf osmotic potential increased to 8% under 400 mM NaCl, which is close to the contribution of K^+^ (**Table 1**). These results suggest that the betaine performed OA in *A. canescens* seedlings under higher salinity. On the other hand, it was reported that the betaine plays other roles in *Atriplex* genus species in response to salinity. For example, Tsutsumi et al. (2015) reported that high salinity induced the accumulation of betaine in the cytosol of the salt bladders of *A. gmelini*, which contributed to maintain membrane integrity and the enzyme activity and, as a result, ensured the bladder cells to load Na^+^ into vacuole. In *A. halimus*, the accumulation of betaine helps to protect the photosynthetic machinery from salinity (Ben Hassine et al., 2008). Moreover, previous studies proposed that free proline is involved in the response to drought stress rather than to salinity in *A. halimus* (Ben Hassine et al., 2008, 2009). In *A. canescens*, however, the accumulation of leaf free proline was strongly induced by high salinity (200 and 400 mM NaCl) though it was unaffected by moderate concentrations (100 mM) of NaCl (**Figure 7B**), suggesting that the free proline may also be involved in physiological response of *A. canescens* to high salinity.

## Conclusion

Our results demonstrate that the growth of *A. canescens* can be stimulated by moderate salinity (100 mM NaCl) and was not inhibited by higher salinity (200 and 400 mM NaCl). This adaptation is achieved through the following aspects: (i) to enhance the photosynthetic capacity by improving Pn and WUE. (ii) to increase Na^+^ accumulation in tissues and salt bladders, as well as improve transport capacity for K^+^ over Na^+^ (ST value) from stem to leaf, which may maintain intracellular K^+^ homeostasis. (iii) to maintain OA capacity and improve the water status in plant by accumulation of inorganic ions and compatible solutes.

## Author Contributions

Y-QP, S-MW, and A-KB conceived the study and designed the experiments; Y-QP and HG performed most of the work; BZ, J-LZ, H-JY, and QM provided technical assistance to experiments and data analysis, as well as, made revisions on the article. S-MW gave valuable suggestions on the article. Y-QP and A-KB wrote the article.

## Conflict of Interest Statement

The authors declare that the research was conducted in the absence of any commercial or financial relationships that could be construed as a potential conflict of interest.
